# Identification of Novel Plasma Biomarkers for Abdominal Aortic Aneurysm by Protein Array Analysis

**DOI:** 10.3390/biom12121853

**Published:** 2022-12-12

**Authors:** Jianqiang Wu, Wei Wang, Ting Xie, Zhaoran Chen, Lei Zhou, Xiaohong Song, Haoxuan Kan, Yanze Lv, Lianglin Wu, Fangda Li, Dan Yang, Yuexin Chen, Bao Liu, Yuehong Zheng

**Affiliations:** 1 State Key Laboratory of Complex Severe and Rare Disease, Department of Medical Research Center, Peking Union Medical College Hospital, Chinese Academy of Medical Sciences and Peking Union Medical College, Beijing 100730, China; 2 Department of Vascular Surgery, Beijing Friendship Hospital, Capital Medical University, Beijing 100050, China; 3 Department of Geriatrics and Gerontology, Beijing Friendship Hospital, Capital Medical University, Beijing 100050, China; 4 Department of Vascular Surgery, Peking Union Medical College Hospital, Chinese Academy of Medical Sciences and Peking Union Medical College, Beijing 100730, China; 5 Department of Computational Biology and Bioinformatics, Institute of Medicinal Plant Development, Chinese Academy of Medical Sciences and Peking Union Medical College, Beijing 100193, China

**Keywords:** abdominal aortic aneurysm, biomarker discovery, protein array analysis, type 2 diabetes, therapeutic targets

## Abstract

Abdominal aortic aneurysm (AAA) is a potentially life-threatening disease that is common in the aging population. Currently, there are no approved diagnostic biomarkers or therapeutic drugs for AAA. We aimed to identify novel plasma biomarkers or potential therapeutic targets for AAA using a high-throughput protein array-based method. Proteomics expression profiles were investigated in plasma from AAA patients and healthy controls (HC) using 440-cytokine protein array analysis. Several promising biomarkers were further validated in independent cohorts using enzyme-linked immunosorbent assay (ELISA). Thirty-nine differentially expressed plasma proteins were identified between AAA and HC. Legumain (LGMN) was significantly higher in AAA patients and was validated in another large cohort. Additionally, “AAA without diabetes” (AAN) patients and “AAA complicated with type 2 diabetes mellitus” (AAM) patients had different cytokine expression patterns in their plasma, and nine plasma proteins were differentially expressed among the AAN, AAM, and HC subjects. Delta-like protein 1 (DLL1), receptor tyrosine-protein kinase erbB-3 (ERBB3), and dipeptidyl peptidase 4 (DPPIV) were significantly higher in AAM than in AAN. This study identified several promising plasma biomarkers of AAA. Their role as therapeutic targets for AAA warrants further investigation.

## 1. Introduction

Abdominal aortic aneurysm (AAA) is a common and potentially life-threatening disease. It is defined as permanent enlargement of the aortic wall greater than 1.5 times the normal adjacent aortic diameter [1]. The prevalence of AAA in individuals over the age of 65 is 4–7% in men and 1–2% in women; AAA is the 13th leading cause of death in the United States [2]. The main complication of AAA is aneurysm rupture, which causes approximately 200,000 deaths each year worldwide [3]. AAA is usually asymptomatic until rupture occurs. To evaluate the risk of AAA rupture early, ultrasound screening is recommended for people over 65 years of age in several countries [4,5]. However, no biomarker that can serve as a screening and surveillance tool for AAA in clinical practice has been made available to date.

AAAs with a diameter greater than 5.5 cm are referred for open surgical repair or endovascular aneurysm repair, while small asymptomatic AAAs are recommended for conservative monitoring of the aneurysm’s diameter [6,7]. However, because the pathogenesis of AAA is not well understood, there are no available therapeutic drugs to prevent the development or rupture of AAA [8]. The main pathological features of AAA include extracellular matrix (ECM) remodeling, loss of vascular smooth muscle cells (VSMCs), inflammation and immune response, intraluminal thrombus formation, and oxidative stress [9,10]. Type 2 diabetes mellitus (T2DM) is a traditional risk factor for multiple cardiovascular diseases. However, epidemiological studies have revealed a negative relationship between T2DM and the morbidity, growth rate, and rupture of AAA, but whether there is a difference between diabetic and nondiabetic patients in terms of prognosis after AAA treatment is controversial [11,12,13,14]. T2DM is becoming known as a potentially protective factor against AAA. However, the exact molecular mechanisms involved in the inverse association between T2MD and AAA are still unknown. The identification of biomarkers associated with AAA may contribute to revealing the possible pathophysiologic mechanism and discovering potential therapeutic targets for AAA.

Proteomics is a powerful tool for biomarker discovery, pathogenesis exploration, and drug target identification. We hypothesized that certain promising biomarkers or therapeutic targets could be identified in the plasma samples of AAA patients using high-throughput antibody array-based proteomics analysis. In this study, we performed 440-cytokine protein array analysis of plasma samples from AAA patients and healthy controls (HC) and aimed to identify novel diagnostic biomarkers of AAA. Meanwhile, we also compared cytokine expression profiling between “AAA without diabetes” (AAN) patients and “AAA complicated with T2DM” (AAM) patients. We aimed to explore the potential protective mechanism of T2DM against AAA and to identify potential therapeutic targets for AAA.

## 2. Materials and Methods

### 2.1. Patients and Sample Collection

Consecutive AAA patients were recruited from Peking Union Medical College Hospital (PUMCH). AAA was diagnosed using computed tomography angiography; patients with an aortic diameter of ≥30 mm were considered candidates for the study. The exclusion criteria included inflammatory AAA, dissecting AAA, false aneurysms, ruptured aneurysms, Takayasu arteritis, and cancer. AAM patients were selected from among the AAA patients. T2DM patients were identified by their medical history and medication history, and their diabetes duration, fasting glucose, glycosylated hemoglobin, and medication usage were recorded. Healthy controls (HCs) were recruited from the physical examination center of PUMCH during the same period and matched with the age, sex, and several confounding factors (including hypertension, dyslipidemia, and diabetes) of the AAA patients. The exclusion criteria for HCs included infectious diseases, rheumatic immune diseases, and cancer. This study was approved by the Ethics Committee of PUMCH (No. JS-2629), and written consent was obtained from all patients and control participants.

In the biomarker discovery cohort, plasma samples were collected from 22 AAA patients and 10 HCs to identify potential biomarkers of AAA using protein array analysis. AAA patients in this cohort were predominantly male, with an average age of 71 years. Their average aneurysmal diameter was 53.0 mm. In validation cohort 1, 94 AAA patients and 60 HCs were recruited to validate promising biomarkers using ELISA. The average age of the AAA patients in this cohort was 69 years, and their average aneurysmal diameter was 52.6 mm. In validation cohort 2, another 22 AAA patients and 10 HCs were recruited for validation of several key proteins that may be involved in the inverse association between T2MD and AAA using ELISA. The average age of the AAA patients in this cohort was 70 years, and their average aneurysmal diameter was 46.3 mm.

A morning fasting venous blood sample was collected from each subject into an EDTA anticoagulation blood collection tube and centrifuged at 2000 rpm for 15 min. The supernatant was stored at −80 °C for further analysis.

### 2.2. Antibody Array and Data Processing

All 32 plasma samples in the biomarker discovery cohort were analyzed using antibody arrays of 440 cytokines (details in Supplementary Table S1, GSH-CAA-440, Norcross, GA, USA). All experiments were carried out according to the manufacturer’s instructions (www.raybiotech.com/human-cytokine-array-gs440-en/, accessed on 1 October 2021). Briefly, after blocking the antibody array for 1 h at room temperature, 60 µL of threefold-diluted plasma was added to each well, and incubation was conducted at 4 °C overnight. After thorough washing, antibodies labeled with biotin were added for 2 h, then washing was performed again. Next, 80 µL of Cy3-conjugated streptavidin was added, followed by incubation for 1 h at room temperature. After another washing step, the signals were scanned with a microarray scanner (InnoScan 300, Innopsys, Carbonne, France) at the appropriate wavelength for Cy3, and the fluorescence intensity data were analyzed using Q-Analyzer Software (RayBiotech, Peachtree Corners, GA, USA).

Raw data from the array scanner were provided as images (.tif files) and spot intensities (Excel.xls files; Microsoft, Seattle, WA, USA) and were processed using background removal and interchip normalization. Normalization data were further analyzed, and the differentially expressed proteins in different groups were defined as those proteins with a fold change >1.5 or <0.67 relative to the control group (*p*-value < 0.05 (*t*-test) and fluorescent value > 150).

### 2.3. Bioinformatics Analysis

A volcano plot was generated using the OmicSolution website (www.omicsolution.org/wkomics/main/, accessed on 7 June 2022). Orthogonal partial least squares discriminant analysis (OPLS-DA) was conducted using SIMCA 15.0 (Umetrics, Umeå, Sweden) software. Principal component analysis (PCA) was performed with the R package “ggfortify”, and a cluster heatmap was constructed using the R package “gplots”. UniProt numbers of the differential proteins were used as inputs for Ingenuity Pathway Analysis (IPA version 2.3; Qiagen, Redwood City, CA, USA).

### 2.4. ELISA

ELISA kits were utilized to validate the crucial cytokine results according to the manufacturer’s instructions (RayBiotech, Peachtree Corners, GA, USA, Catalog #: ELH-Legumain, ELH-ErbB3, ELH-DLL1, and ELH-CD26). Briefly, plasma samples were diluted with different dilution factors based on individual plasma biomarkers. The samples were then coated on ELISA plates overnight at 4 °C. The plates were subsequently washed with wash buffer, and a biotin-conjugated antibody was added to the ELISA plates, followed by incubation for 1 h. HRP-conjugated streptavidin was added to catalyze the tetramethylbenzidine (TMB) reagent reaction. Finally, the catalytic reaction was stopped by the addition of sulfuric acid. In each incubation step, the volume in each well was 100 μL. Finally, the OD450 was determined using a microplate reader (ELx800NB, Biotek, Winooski, VT, USA). ELISA assays were carried out in duplicate, and the data were represented as the mean of the duplicates.

### 2.5. Statistical Analysis

Data analysis was performed using IBM SPSS Statistics (version 20.0, IBM Corp., Armonk, NY, USA) and GraphPad Prism (version 8.0.2, GraphPad Software, San Diego, CA, USA) software. Categorical variables were analyzed using the chi-square test or Fisher’s exact test. Continuous variables were compared using Student’s *t*-test or the Mann–Whitney U test. Comparisons among the three groups were conducted using one-way ANOVA followed by multiple comparisons. Receiver operating characteristic (ROC) analysis was performed on the MetaAnalyst 5.0 website (www.metaboanalyst.ca/, accessed on 14 June 2022) and MedCalc Statistical Software (version 19.0.4, MedCalc Software bvba, Ostend, Belgium).

## 3. Results

### 3.1. Clinical Characteristics of the Participants and Study Design

A total of 138 AAA patients and 80 HCs were included in this study; these subjects were divided into three cohorts. The clinical characteristics of the subjects are shown in Table 1. Several confounding factors, such as age, sex, hypertension, and dyslipidemia, were matched between AAA and HC or between AAM and AAN. The aneurysmal diameter of the AAN patients was significantly larger than that of the AAM patients, both in the discovery cohort and validation cohort 2 (60 ± 16 vs. 47 ± 10 mm, *p* = 0.019; 51 ± 8 vs. 41 ± 7 mm, *p* = 0.003, respectively).

To identify potential diagnostic biomarkers of AAA in the discovery cohort, differentially expressed proteins between the 22 AAA patients and 10 HCs were identified via protein array analysis, and further bioinformatics analyses of these differential proteins were performed. Next, promising biomarker candidates were further verified in validation cohort 1 (94 AAA patients and 60 HCs). Then, subgroup analysis of the protein array data was performed to reveal the cytokine expression patterns between AAM and AAN patients. Differential proteins were identified among AAN, AAM, and HCs, and several important plasma proteins were further verified in validation cohort 2 (22 AAA patients and 10 HCs) using ELISA.

### 3.2. Identification of Differentially Expressed Proteins between AAA and HC

**Figure 1:**
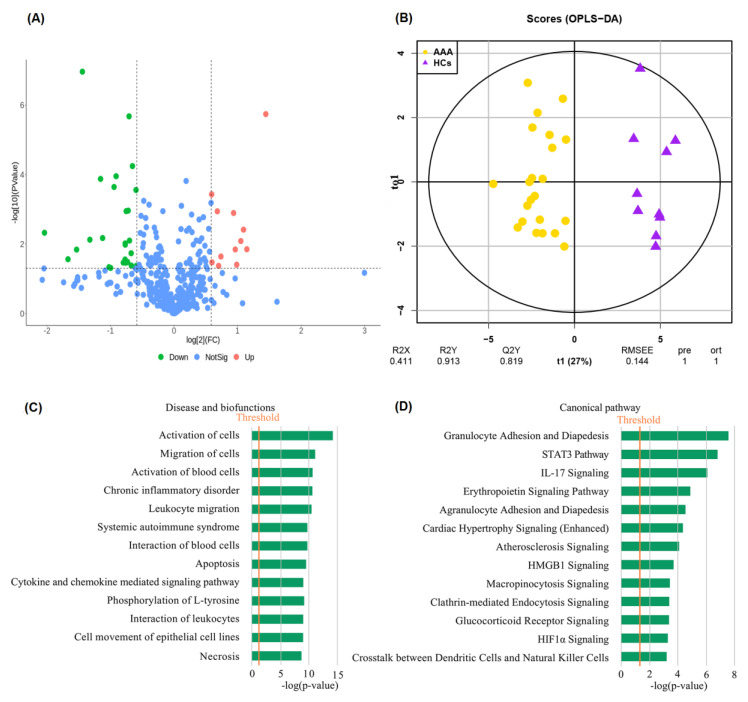
Bioinformatics analysis of differentially expressed proteins between AAA patients and healthy controls. (**A**) Volcano plot of differentially expressed proteins; (**B**) orthogonal partial least squares discriminant analysis (OPLS−DA) result; (**C**) disease and biofunction results from IPA analysis; (**D**) canonical pathway results from IPA analysis.

High-throughput antibody array analysis of the 22 AAA patients and 10 HCs was performed to explore the diagnostic biomarkers of AAA. A total of 39 differentially expressed proteins were identified, of which 12 plasma proteins were upregulated and 27 plasma proteins were downregulated (Figure 1A, Table 2). Meanwhile, OPLS-DA analysis was conducted, and the results showed that protein expression profiles were significantly different between AAA and HC (Figure 1B).

IPA analyses were further performed to reveal the biofunctions and canonical pathways of the differentially expressed proteins associated with AAA. The results showed that in the disease and biofunction category, the differential proteins were enriched in activation of cells, migration of cells, activation of blood cells, chronic inflammatory disorder, leukocyte migration, interaction of blood cells, apoptosis, cytokine- and chemokine-mediated signaling pathway, interaction of leukocytes, cell movement of epithelial cell lines, and necrosis (Figure 1C). Canonical pathway analysis indicated that the differential proteins were enriched in granulocyte adhesion and diapedesis, STAT3 pathway, IL-17 signaling, erythropoietin signaling pathway, agranulocyte adhesion and diapedesis, atherosclerosis signaling, HMGB1 signaling, micropinocytosis signaling, clathrin-mediated endocytosis signaling, glucocorticoid receptor signaling, HIF1α signaling, and crosstalk between dendritic cells and natural killer cells (Figure 1D).

### 3.3. Identification of Potential Diagnostic Biomarkers of AAA

**Figure 2:**
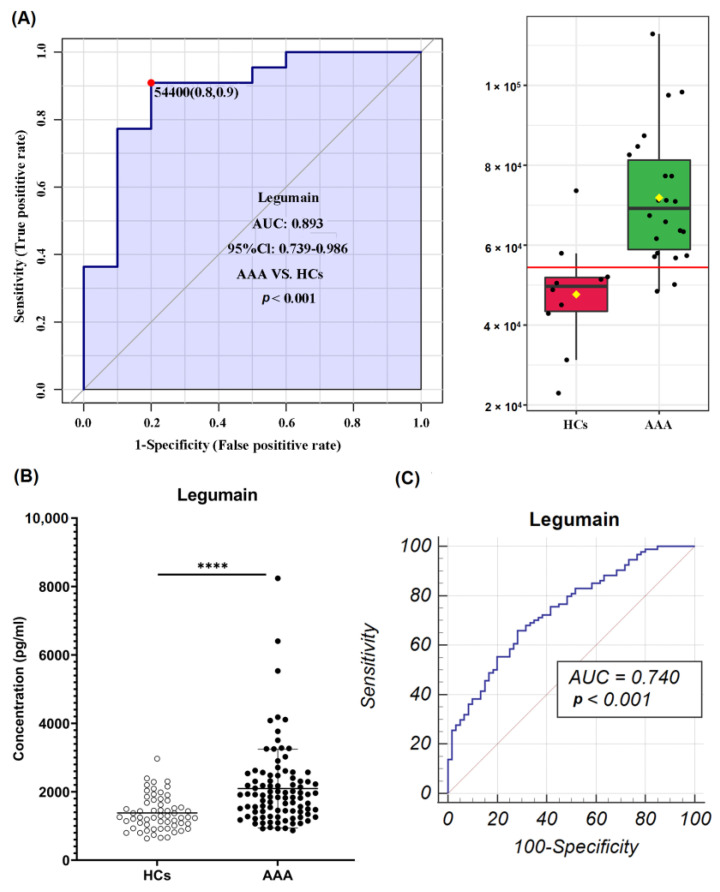
Diagnostic performance of LGMN in the discovery and validation cohorts. (**A**) ROC analysis of LGMN according to protein array data; (**B**) ELISA result of LGMN in validation cohort 2; (**C**) ROC analysis of LGMN in validation cohort 2. **** *p* < 0.0001.

To identify potential diagnostic biomarkers of AAA, ROC analysis was performed for the 39 differentially expressed proteins; it identified 13 plasma proteins with a large area under the curve (AUC) value (>0.85) (Supplementary Table S2). Combining ROC analysis and literature retrieval results, Legumain (LGMN) was selected as the key protein that could serve as a novel diagnostic biomarker of AAA, with an AUC of 0.893 (95% CI: 0.739–0.986, *p* < 0.001) (Figure 2A).

To further explore the diagnostic value of LGMN, we performed ELISA validation in a larger cohort. The ELISA result was consistent with the protein array result: LGMN was highly expressed in the AAA group compared with the HC group (Figure 2B). ROC analysis showed that LGMN had a high value in diagnosing AAA, with an AUC value of 0.740 (95% CI: 0.663–0.807, sensitivity: 65.95, specificity: 71.67) (Figure 2C).

### 3.4. Exploration of Cytokine Expression Patterns in AAN and AAM Patients

**Figure 3:**
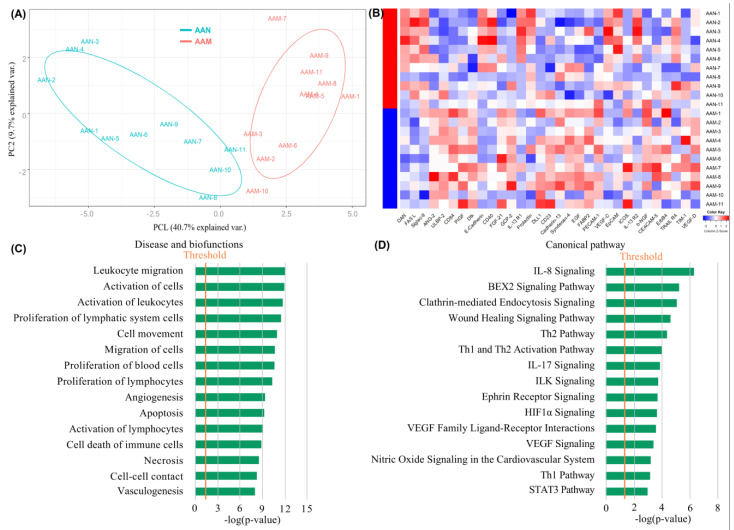
Identification and bioinformatics analyses of differentially expressed proteins between AAM and AAN patients. (**A**) Principal component analysis (PCA) model between AAM and AAN; (**B**) Clustering heatmap of differential proteins between the two groups; (**C**) Disease and biofunction analysis; (**D**) Canonical pathway analysis.

To explore the biomarkers that could potentially be related to the inverse association between T2DM and AAA, we conducted subgroup analysis of the protein array data and investigated the cytokine expression patterns of AAN and AAM patients. As a result, 31 differentially expressed proteins were identified between the AAN and AAM groups, of which 11 plasma proteins were upregulated and 20 plasma proteins were downregulated (Supplementary Table S3). Moreover, apparent differences between the AAN and AAM groups were observed via OPLS-DA analysis (Figure 3A). The clustering heatmap also showed that plasma cytokines were significantly different between the two groups (Figure 3B).

IPA analysis revealed that in the disease and biofunction category, the differential proteins were significantly enriched in leukocyte migration, activation of cells, activation of leukocytes, proliferation of lymphatic system cells, cell movement, migration of cells, proliferation of blood cells, proliferation of lymphocytes, angiogenesis, apoptosis, activation of lymphocytes, cell death of immune cells, and necrosis (Figure 3C). In the canonical pathway category, the differential proteins were mainly associated with IL-8 signaling, the BEX2 signaling pathway, clathrin-mediated endocytosis signaling, the wound healing signaling pathway, the Th1 and Th2 pathways, IL-17 signaling, ILK signaling, ephrin receptor signaling, and HIF1α signaling (Figure 3D).

### 3.5. Identification of Differentially Expressed Proteins among AAM, AAN, and HC

Further analysis of plasma proteins among AAM, AAN, and HC identified nine important proteins in three groups: (1) differentially expressed between AAN and HCs; (2) differentially expressed between AAM and AAN; and (3) showing an opposite trend between (1) and (2). These proteins included delta-like protein 1 (DLL1), dipeptidyl peptidase 4 (DPPIV), receptor tyrosine-protein kinase erbB-3 (ERBB3), interleukin-18 receptor 1 (IL-1 R5), serine protease 27 (marapsin), placental growth factor (PIGF), prostasin, RGM domain family member B (RGM-B), and tumor necrosis factor receptor superfamily member 27 (XEDAR). Previous studies revealed that type 2 diabetes (T2DM) might offer protection against the development and progression of AAA. Moreover, the aneurysmal diameter of the AAM patients in our study was significantly smaller than that of the AAN patients. Thus, these proteins may be involved in the pathophysiological mechanisms underlying the inverse association between T2DM and AAA.

**Figure 4:**
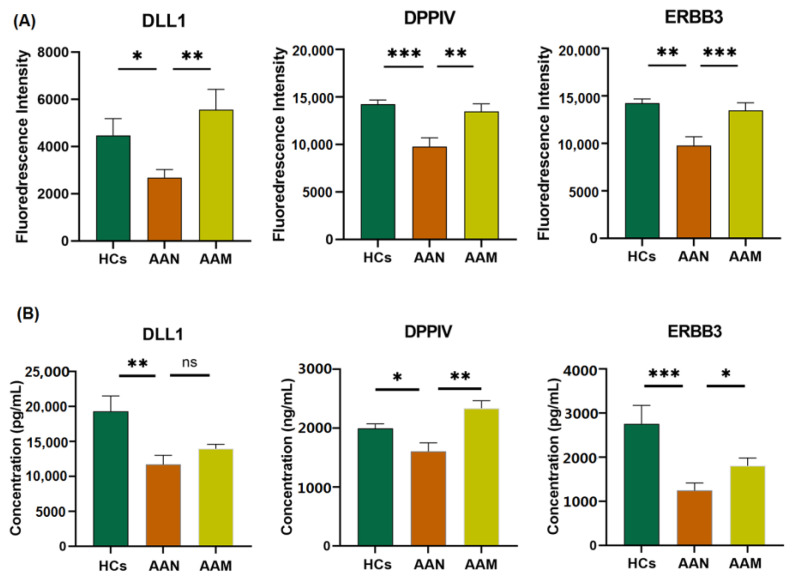
Exploration of crucial proteins among AAM and AAN patients and healthy controls. (**A**) Protein array results; (**B**) ELISA results in validation cohort 2. Data represent mean ± standard error. One-way ANOVA followed by multiple comparisons was conducted to examine the differences among the three groups. * *p* < 0.05, ** *p* < 0.01, *** *p* < 0.001, ns: Not significant. DLL1: delta-like protein 1, DPPIV: dipeptidyl peptidase 4, ERBB3: receptor tyrosine-protein kinase erbB-3.

Using literature retrieval, we searched for possible relationships between these proteins and AAA or T2DM. Based on the known pathophysiological process of AAA and T2DM, we selected three important proteins (DLL1, DPPIV, and ERBB3) for further validation (Figure 4). To validate the protein array data, DLL1, DPPIV, and ERBB3 were analyzed by ELISA in the plasma samples of another independent patient cohort (validation cohort 2). The results were consistent with the protein array data. DLL1, DPPIV, and ERBB3 were significantly downregulated in AAN patients compared with HCs. On the other hand, they were significantly upregulated in AAM compared with AAN, and their expression levels returned to levels similar to those of HCs. These results indicated that DLL1, ERBB3, and DPPIV have the potential to serve as therapeutic targets of AAA.

## 4. Discussion

AAA is a common and potentially life-threatening disease. However, no approved disease biomarkers or therapeutic drugs are available for AAA in clinical practice. Recently, several promising plasma biomarkers have been proposed for the clinical management of AAA, such as myeloperoxidase, D-dimer, GDF 15, cystatin B, and attractin [15,16,17]. Whether these biomarkers can be used for disease screening, monitoring, or therapeutic decision making warrants further investigation. In the current study, we aimed to identify novel plasma biomarkers or therapeutic targets for AAA using a high-throughput human cytokine antibody array. This array can simultaneously and quantitatively detect 440 human cytokines in a single experiment. Moreover, low-abundance proteins, such as growth factors and cytokines, are difficult to detect using mass spectrometry-based proteomic methods. An antibody-based protein array is a complementary approach for reliably quantifying these proteins [18]. In this study, 39 differentially expressed proteins were identified between the AAA and HC groups, and the levels of nine plasma proteins were significantly different among AAN, AAM, and HC.

To better understand the 39 differentially expressed proteins between AAA and HC, bioinformatics analysis was performed using IPA. The results showed that immuno-inflammatory-related pathways were significantly enriched in AAA, including granulocyte adhesion and diapedesis, the STAT3 pathway, IL-17 signaling, agranulocyte adhesion and diapedesis, HMGB1 signaling, and crosstalk between dendritic cells and natural killer cells. Previous studies have shown that inhibition of the STAT3 pathway attenuated experimental AAA progression [19]. IL-17 signaling has been reported to be activated in AAA patients, and it could facilitate experimental AAA formation, which may be associated with the promotion of inflammation [20]. HMGB1 is a danger signal that can mediate cross talk between immune cells, resulting in inflammation and cell injury; inhibiting HMGB1 signaling attenuated AAA progression in mice [21]. Meanwhile, the erythropoietin (EPO) signaling pathway and HIF1α signaling were significantly enriched in AAA. A recent study demonstrated that EPO was associated with AAA, which could promote experimental AAA formation by increasing angiogenesis, inflammation, collagen degradation, and apoptosis of VSMCs [22]. In addition, another previous study suggested a promotive role for HIF1β in AAA development [23]. Interestingly, micropinocytosis signaling and clathrin-mediated endocytosis signaling were significantly enriched in AAA. This phenomenon requires further study.

Among the 39 differential proteins between AAA and HC, 13 cytokines showed high AUC values. Clusterin, MMP-7, junctional adhesion molecule A (JAM-A), cathepsin S, angiotensin-converting enzyme 2 (ACE-2), C–C motif chemokine 5 (RANTES), and oncostatin-M (OSM) have previously been reported to be associated with AAA development, reflecting the pathophysiological changes in AAA. LGMN, RGM-B, erythropoietin receptor (EpoR), interleukin-1 receptor type 2 (IL-1R2), thrombopoietin (TPO), and proneuregulin-1 (NRG1-b1) have seldom been reported to be related to AAA development, according to a literature review. According to the pathogenesis of AAA, LGMN was selected and further validated in a larger cohort.

LGMN can induce the activation of MMP-2 and MMP-9, which are associated with ECM degradation [24]. ECM degradation is considered one of the most important pathophysiological processes leading to AAA, and MMPs are crucial factors in this process [25]. Meanwhile, Solberg et al. revealed that LGMN expression, activity, and secretion were related to macrophage differentiation [26]. Macrophage differentiation has a significant effect on the development of AAA [10]. Although it has not been reported whether LGMN can promote AAA development, a recent study discovered that LGMN was significantly upregulated in the aorta and serum of thoracic aortic dissection (TAD) patients. Moreover, TAD development was significantly inhibited in LGMN-deficient mice. Mechanistically, macrophage-derived LGMN may promote TAD development by regulating VSMC differentiation. Thus, LGMN may be a novel target for the prevention and treatment of TAD [27]. In our protein array analysis, LGMN was significantly upregulated in the plasma of AAA patients relative to HCs. Further ELISA analysis in a larger cohort validated the diagnostic value of LGMN. Therefore, LGMN may be a novel and promising biomarker or treatment target for AAA.

Interestingly, epidemiologic studies have observed an inverse association between T2DM and AAA [14]. T2DM is beginning to be seen as a potentially protective factor against AAA. However, the molecular mechanisms of this protective effect remain unclear. Previous studies have suggested that possible mechanisms may include decreased ECM remodeling, decreased inflammation and oxidative stress, increased glycation and advanced glycation end products, intraluminal thrombus biology, neoangiogenesis, and VSMC preservation. In addition, some studies have reported that antidiabetic treatments might be negatively related to AAA formation [28,29,30]. In our study, we analyzed drug management in AAM patients and investigated the effect of medications on the differentially expressed proteins. However, no hypoglycemic drugs showed an association with the differential proteins. To explore the possible mechanism underlying the inverse association between T2DM and AAA, we conducted a subgroup analysis of the protein array data. The results showed different cytokine expression patterns between AAM and AAN patients. ELISA analysis further supported the potential roles of several key proteins in AAA, including DLL1, DPPIV, and ERBB3.

DLL1 functions as a transmembrane ligand protein of Notch receptors and triggers Notch signaling [31]. DLL1 protein levels were reported to be significantly lower in descending thoracic aortic aneurysm and dissection tissues [32], but the function of DLL1 in AAA is still unknown. Notch signaling was reported to be downregulated in human aneurysmal tissue [33,34]. Rubey et al. found that DLL1 was specifically expressed in pancreatic β-cells and that overexpression of DLL1 caused impaired glucose tolerance and reduced insulin secretion [35]. In addition, in some diseases associated with diabetes, DLL1 or Notch signaling was found to be more highly expressed in some organs [36,37]. Our study showed that DLL1 was expressed at lower levels in AAN but at higher levels in AAM, which is supported by some previous studies. These results suggest that DLL1 may be a potential therapeutic target for AAA that deserves further investigation.

DPPIV is a serine protease that exists as a membrane-bound cell surface peptidase and can also occur in the circulation in a soluble form [38]. A recent study showed that DPPIV levels were elevated in both the media and adventitia of human AAA tissue, which was also correlated with the expression of genes related to the pathophysiological processes of AAA, such as inflammation, proteolysis, and apoptosis. In addition, a lower plasma DPPIV level was detected in AAA patients relative to control subjects [39]. Animal studies have suggested that DPPIV inhibitors can suppress the development and progression of AAA [40]. Additionally, one of the well-known functions of DPPIV is to degrade incretins, which can promote insulin secretion from pancreatic β-cells and lower blood glucose levels. The inhibition of DPPIV can attenuate hyperglycemia by increasing incretin availability and insulin levels. As a result, DPPIV inhibitors have been developed as a new class of drugs for the clinical treatment of T2DM [41]. Moreover, most studies have supported the finding of elevated plasma DPPIV activity and/or levels in patients with diabetes [42]. DPPIV is considered an important predictor of the onset of insulin resistance [43]. Our data revealed differences in plasma DPPIV levels that are consistent with previous studies. Thus, DPPIV is related to the pathogenesis of AAA, and further studies are needed to determine the underlying mechanism.

The ERBB3 heterodimer is considered a potent activator of the PI3K/AKT pathway [44,45]. However, until now, there have been no investigations about the relationship between ERBB3 and the pathogenesis of AAA. The role of PI3K/AKT signaling in the pathophysiology of AAA has been investigated. It has been reported that the activation of PI3K/AKT signaling could attenuate AAA formation by suppressing inflammation and reducing the loss of VSMCs [46,47]. Additionally, some studies have indicated that the gene polymorphism of ERBB3 is closely associated with diabetes [48]. The stress-dependent transactivation of ERBB2–ERBB3 receptors induces serine phosphorylation in insulin receptor substrate 1, which leads to insulin resistance in liver cells [49]. Stress-induced p38MAPK activation leading to the activation of ERBB3 was shown to be related to insulin resistance in a mouse model [50]. Thus, the ERBB signaling pathway might represent a promising research target in AAA development.

In summary, four promising biomarkers (LGMN, DLL1, ERBB3, and DPPIV) that have the potential to serve as diagnostic biomarkers or therapeutic targets for AAA were discovered in this study. A limitation of this study is its relatively small sample size of AAM patients. Due to the potential protective effect of T2DM against the occurrence and development of AAA, it is difficult to recruit AAM patients, and only 22 AAM patients were enrolled in this study. In the discovery stage, we chose 11 AAM, 11 AAN, and 10 HC samples to perform the high-throughput protein array experiment. Then, we selected another 11 AAM, 11 AAN, and 10 HC samples for ELISA validation of DLL1, ERBB3, and DPPIV. In the future, a large-sample, multicenter, prospective study will be necessary to contribute to clinical practice and etiological studies focusing on AAA. Further studies aimed at uncovering the molecular mechanism by which T2DM is inversely associated with AAA are needed.

## 5. Conclusions

In this study, we performed a high-throughput protein array study using the plasma of AAA patients and HCs. Some potential biomarkers of AAA were identified. LGMN is a novel biomarker of AAA with high diagnostic performance. Meanwhile, a different protein expression pattern was identified between the plasma of the AAM and AAN patients. DLL1, ERBB3, and DPPIV may participate in the underlying mechanism of the inverse association between T2DM and AAA. These promising biomarkers were also validated in the plasma of two independent cohorts, indicating their potential to serve as diagnostic biomarkers or therapeutic targets for AAA.

## Figures and Tables

**Table 1:** Clinical characteristics of the subjects in this study.

Variable	Discovery Cohort				Validation Cohort 1			Validation Cohort 2			
	HC	AAN	AAM	*p*-Value	HC	AAA	*p*-Value	HC	AAN	AAM	*p*-Value
Number	10	11	11	—	60	94	—	10	11	11	—
Sex (male/female)	8/2	10/1	10/1	0.688	51/9	81/13	0.819	8/2	10/1	10/1	0.688
Age (years)	68 ± 9	69 ± 8	73 ± 8	0.309	68 ± 7	69 ± 10	0.438	67 ± 3	69 ± 7	70 ± 7	0.443
Diameter (mm)	—	60 ± 16	47 ± 10	0.019	—	53 ± 13	—	—	51 ± 8	41 ± 7	0.003
Hypertension	4	7	7	0.458	39	73	0.085	7	8	7	0.895
Type 2 Diabetes	0	0	11	—	11	17	0.969	0	0	11	—
Dyslipidemia	3	8	7	0.119	28	58	0.071	4	8	7	0.293

HC, healthy controls; AAA, abdominal aortic aneurysm; AAN, AAA patients not complicated with diabetes; AAM, AAA patients complicated with type 2 diabetes. Comparison of different groups was performed using analysis of variance (ANOVA), Student’s *t*-test, or the chi-square test. Data are presented as number or mean ± standard deviation. A *p*-value < 0.05 is indicated as statistically significant.

**Table 2:** Differentially expressed proteins between AAA patients and healthy controls using protein array analysis.

Gene Symbol	UniPort ID	Ratio (AAA/HCs)	*p*-Value
MMP-7	P09237	2.72	1.82 × 10^−6^
Clusterin	P10909	0.37	1.09 × 10^−7^
RGM-B	Q6NW40	0.61	2.11 × 10^−6^
Legumain	Q99538	1.51	0.0003
Epo R	P19235	0.64	5.72 × 10^−5^
JAM-A	Q9Y624	1.92	0.0013
NRG1-b1	Q02297	0.52	0.0002
Cathepsin S	P25774	0.66	0.0003
DAN	P41271	0.61	0.0011
OSM	P13725	0.24	0.0046
IL-13 R1	P78552	0.59	0.0011
ACE-2	Q9BYF1	0.53	0.0001
IL-1 RII	P27930	0.45	0.0001
IL-13 R2	Q14627	0.40	0.0075
RANTES	P13501	1.60	0.0011
FAS L	P48023	0.62	0.0080
MICB	Q29980	0.59	0.0096
TARC	Q92583	2.14	0.0039
TPO	P40225	0.59	0.0348
Galectin-7	P47929	0.31	0.0276
Syndecan-3	O75056	0.35	0.0145
ULBP-2	Q9BZM5	2.07	0.0082
E-Cadherin	P12830	0.66	0.0425
GH 1	P01241	0.59	0.0105
TRAIL R4	Q9UBN6	0.59	0.0332
GITR L	Q9UNG2	0.49	0.0465
B7-1	P33681	0.63	0.0185
LAP (TGFb1)	P01137	1.95	0.0144
ADAMTS13	Q76LX8	0.57	0.0345
PECAM-1	P16284	1.67	0.0230
GROa	P09341	0.46	0.0068
FABP2	P12104	1.63	0.0427
ANG-4	Q9Y264	0.60	0.0332
Granulysin	P22749	0.59	0.0280
PDGF-AB	Q08CD2	2.21	0.0142
WISP-1	Q96FT7	0.63	0.0420
EGF	P01133	1.98	0.0396
PDGF-BB	P01127	1.51	0.0339
BAFF	Q9Y275	0.50	0.0491

HC, healthy controls; AAA, abdominal aortic aneurysm.

## Data Availability

The data presented in this study are available on request from the corresponding author. The data are not publicly available due to the small possibility of compromising the individual privacy of patients.

## Supplementary Materials

The following supporting information can be downloaded at https://www.mdpi.com/article/10.3390/biom12121853/s1: Table S1: The 440 cytokines of the antibody arrays; Table S2: Differentially expressed proteins between AAA patients and healthy controls with large area under the curve (AUC) values (AUC > 0.85); Table S3: Differentially expressed proteins between AAM and AAN patients.
