# Supplementary material for: Identification of Novel Plasma Biomarkers for Abdominal Aortic Aneurysm by Protein Array Analysis
Source: Biomolecules. 2022 Dec 12;12(12):1853. doi: 10.3390/biom12121853 (PMC9775419; doi:10.3390/biom12121853)
Supplement: Supplementary file 1 [file biomolecules-12-01853-s001.zip › Table S1.pdf]

Supplementary Table S1. The 440 cytokines of the antibody arrays.

Official Gene Symbol	UniProt ID	Entrez Gene ID
4-1BB	Q07011	3604
ALCAM	Q13740	214
B7-1	P33681	941
BCMA	Q02223	608
CD14	P08571	929
CD30	P28908	943
CD40L	P29965	959
CEACAM-1	P13688	634
DR6	O75509	27242
Dtk	Q06418	7301
Endoglin	P17813	2022
ErbB3	P21860	2065
E-Selectin	P16581	6401
Fas	P25445	355
Flt-3L	P36888	2322
GITR	Q9Y5U5	8784
HVEM	Q92956	8764
ICAM-3	P32942	3385
Contactin-2	Q02246	6900
IL-1 RI	P14778	3554
IL-2 Rg	P31785	3561
IL-10 Rb	Q08334	3588
IL-17R	Q96F46	23765
IL-21R	Q9HBE5	50615
LIMPII	Q14108	950
Lipocalin-2	P80188	3934
L-Selectin	P14151	6402

LYVE-1	Q9Y5Y7	10894
MICA	Q29983	100507436
MICB	Q29980	4277
NRG1-b1	Q02297	3084
PDGF Rb	P09619	5159
PECAM-1	P16284	5175
RAGE	Q15109	177
TIM-1	Q96D42	26762
TRAIL R3	O14798	8794
Trappin-2	P19957	5266
uPAR	Q03405	5329
VCAM-1	P19320	7412
XEDAR	Q9HAV5	60401
BLC	O43927	10563
Eotaxin	P51671	6356
Eotaxin-2	O00175	6369
G-CSF	P09919	1440
GM-CSF	P04141	1437
I-309	P22362	6346
ICAM-1	P05362	3383
IFNg	P01579	3458
IL-1a	P01583	3552
IL-1b	P01584	3553
IL-1ra	P18510	3557
IL-2	P60568	3558
IL-4	P05112	3565
IL-5	P05113	3567
IL-6	P05231	3569
IL-6R	P08887	3570

IL-7	P13232	3574
IL-8	P10145	3576
IL-10	P22301	3586
IL-11	P20809	3589
IL-12p40	P29460	3593
IL-12p70	P29459	3592
IL-13	P35225	3596
IL-15	P40933	3600
IL-16	Q14005	3603
IL-17	Q16552	3605
MCP-1	P13500	6347
MCSF	P09603	1435
MIG	Q07325	4283
MIP-1a	P10147	6348
MIP-1b	P13236	6351
MIP-1d	Q16663	6359
PDGF-BB	P01127	5155
RANTES	P13501	6352
TIMP-1	P01033	7076
TIMP-2	P16035	7077
TNFa	P01374	4049
TNFb	Q06643	4050
TNF RI	P19438	7132
TNF RII	P20333	7133
AR	P15514	374
BDNF	P23560	627
bFGF	P09038	2247
BMP-4	P12644	652
BMP-5	P22003	653

BMP-7	P18075	655
b-NGF	P01138	4803
EGF	P01133	1950
EGF R	P00533	1956
EG-VEGF	P58294	84432
FGF-4	P08620	2249
FGF-7	P21781	2252
GDF-15	Q99988	9518
GDNF	P39905	2668
GH	P01241	2688
HB-EGF	Q99075	1839
HGF	P14210	3082
IGFBP-1	P08833	3484
IGFBP-2	P18065	3485
IGFBP-3	P17936	3486
IGFBP-4	P22692	3487
IGFBP-6	P24592	3489
IGF-1	P05019	3479
Insulin	P01308	3630
MCSF R	P07333	1436
NGF R	P08138	4804
NT-3	P20783	4908
NT-4	P34130	4909
OPG	O00300	4982
PDGF-AA	P04085	5154
PIGF	P49763	5228
SCF	P21583	4254
SCF R	P10721	3815
TGFa	P01135	7039

TGFb1	P01137	7040
TGFb3	P10600	7043
VEGF	P15692	7422
VEGF R2	P35968	3791
VEGF R3	P35916	2324
VEGF-D	O43915	2277
6Ckine	O00585	6366
Axl	P30530	558
BTC	P35070	685
CCL28	Q9NRJ3	56477
CTACK	Q9Y4X3	10850
CXCL16	Q9H2A7	58191
ENA-78	P42830	6374
Eotaxin-3	Q9Y258	10344
GCP-2	P80162	6372
GRO	P09341	2919
HCC-1	Q16627	6358
HCC-4	O15467	6360
IL-9	P15248	3578
IL-17F	Q96PD4	112744
IL-18 BPa	O95998	10068
IL-28A	Q8IZJ0	282616
IL-29	Q8IU54	282618
IL-31	Q6EBC2	386653
IP-10	P02778	3627
I-TAC	O14625	6373
LIF	P15018	3976
LIGHT	O43557	8740
Lymphotactin	P47992	6375

MCP-2	P80075	6355
MCP-3	P80098	6354
MCP-4	Q99616	6357
MDC	O00626	6367
MIF	Q07325	4283
MIP-3a	P78556	6364
MIP-3b	Q99731	6363
MPIF-1	P55773	6368
MSP	P26927	4485
NAP-2	P02775	5473
OPN	P10451	6696
PARC	P55774	6362
PF4	P02776	5196
SDF-1a	P48061	6387
TARC	Q92583	6361
TECK	O15444	6370
TSLP	Q969D9	85480
Activin A	P08476	3624
AgRP	O00253	181
Angiogenin	P03950	283
ANG-1	Q15389	284
Angiostatin	P00747	5340
Cathepsin S	P25774	1520
CD40	P25942	958
Cripto-1	P13385	6997
DAN	P41271	4681
DKK-1	O94907	22943
E-Cadherin	P12830	999
EpCAM	P16422	4072

FAS L	P48023	356
Fcg RIIBC	P31994	2213
Follistatin	P19883	10468
Galectin-7	P47929	3963
ICAM-2	P13598	3384
IL-13 R1	P78552	3597
IL-13 R2	Q14627	3598
IL-17B	Q9UHF5	27190
IL-2 Ra	P01589	3559
IL-2 Rb	P14784	3560
IL-23	Q9NPF7	51561
LAP(TGFb1)	P01137	7040
NrCAM	Q92823	4897
PAI-1	P05121	5054
PDGF-AB	Q08CD2	767790
Resistin	Q9HD89	56729
SDF-1b	P48061	6387
gp130	P40189	3572
Shh-N	Q15465	6469
Siglec-5	O15389	8778
ST2	Q01638	9173
TGFb2	P61812	7042
Tie-2	Q02763	7010
TPO	P40225	7066
TRAIL R4	Q9UBN6	8793
TREM-1	Q9NP99	54210
VEGF-C	P49767	7424
VEGF R1	P17948	2321
Adiponectin	Q15848	9370

Adipsin	P00746	1675
AFP	P02771	174
ANGPTL4	Q9BY76	51129
B2M	P61769	567
BCAM	P50895	4059
CA125	Q8WXI7	94025
CA15-3	P15941	4582
CEA	P06731	1048
CRP	P02741	1401
ErbB2	P04626	2064
Ferritin	P02792	2512
FSH	P01225	2488
GROa	P09341	2919
hCGb	P01233	1082
IGF-1R	P08069	3480
IL-1 RII	P27930	7850
IL-3	P08700	3562
IL-18 Rb	O95256	8807
IL-21	Q9HBE4	59067
Leptin	P41159	3952
MMP-1	P03956	4312
MMP-2	P08253	4313
MMP-3	P08254	4314
MMP-8	P22894	4317
MMP-9	P14780	4318
MMP-10	P09238	4319
MMP-13	P45452	4322
NCAM-1	P13591	4684
Nidogen-1	P14543	4811

NSE	P09104	2026
OSM	P13725	5008
Procalcitonin	P06881	796
Prolactin	P01236	5617
PSA-free	P07288	354
Siglec-9	Q9Y336	27180
TACE	P78536	6868
Thyroglobulin	P01266	7038
TIMP-4	Q99727	7079
TSH	P01222	7252
2B4	Q9BZW8	51744
ADAM9	Q13443	8754
ANG-2	O15123	285
APRIL	O75888	8741
BMP-2	P12643	650
BMP-9	Q9UK05	2658
C5a	P01031	727
Cathepsin L	P07711	1514
CD200	P41217	4345
CD97	P48960	976
Chemerin	Q99969	5919
DcR3	O95407	8771
FABP2	P12104	2169
FAP	Q12884	2191
FGF-19	O95750	9965
Galectin-3	P17931	3958
HGF R	P08581	4233
IFNab R2	P48551	3455
IGF-2	P01344	3481

IGF-2R	P11717	3482
IL-1 R6	Q9HB29	8808
IL-24	Q13007	11009
IL-33	O95760	90865
Kallikrein 14	Q9P0G3	43847
Legumain	Q99538	5641
LOX-1	P78380	4973
MBL	P11226	4153
Neprilysin	P08473	4311
Notch-1	P46531	4851
NOV	P48745	4856
Osteoactivin	Q14956	10457
PD-1	Q15116	5133
PGRP-S	O75594	8993
Serpin A4	P29622	5267
sFRP-3	Q92765	2487
Thrombomodulin	P07204	7056
TLR2	O60603	7097
TRAIL R1	O00220	8797
Transferrin	P02787	7018
WIF-1	Q9Y5W5	11197
ACE-2	Q9BYF1	59272
Albumin	P02768	213
AMICA	Q86YT9	120425
ANG-4	Q9Y264	51378
BAFF	Q9Y275	10673
CA19-9		
CD163	Q86VB7	9332
Clusterin	P10909	1191

CRTAM	O95727	56253
CXCL14	O95715	9547
Cystatin C	P01034	1471
Decorin	P07585	1634
Dkk-3	Q9UBP4	27122
DLL1	O00548	28514
Fetuin A	P02765	197
aFGF	P05230	2246
FOLR1	P15328	2348
Furin	P09958	5045
GASP-1	Q8TEU8	124857
GASP-2	Q96NZ8	117166
G-CSF R	Q99062	1441
HAI-2	P20155	6691
IL-17B R	Q9NRM6	55540
IL-27	Q8NEV9	246778
LAG-3	P18627	3902
LDL R	P01130	3949
Pepsinogen I	P0DJD8	643834
RANK	Q9Y6Q6	8792
RBP4	P02753	5950
SOST	Q9BQB4	50964
Syndecan-1	P18827	6382
TACI	O14836	23495
TFPI	P10646	7035
TSP-1	P07996	7057
TRAIL R2	O14763	8795
TRANCE	O14788	8600
Troponin I	P19237	7135

uPA	P00749	5328
VE-Cadherin	P33151	1003
WISP-1	Q96FT7	55515
ANGPTL3	Q9Y5C1	27329
bIG-H3	Q15582	7045
CA9	Q16790	768
Cathepsin B	P07858	1508
CD23	P06734	2208
CHI3L1	P36222	1116
CTLA4	P16410	1493
Dkk-4	Q9UBT3	27121
DPPIV	P27487	1803
EDA-A2	Q92838	1896
Epo R	P19235	2057
FGF-6	P10767	2251
FGF-9	P31371	2254
Gas 1	P54826	2619
IGFBP-5	P24593	3488
IL-1 F5	Q9UBH0	26525
IL-1 F6	Q9UHA7	27179
IL-1 F7	Q9NZH6	27178
IL-1 F8	Q9NZH7	27177
IL-1 F9	Q9NZH8	56300
IL-1 F10	Q8WWZ1	84639
IL-1 R5	Q13478	8809
IL-17C	Q9P0M4	27189
IL-18	Q14116	3606
IL-20	Q9NYY1	50604
IL-34	Q6ZMJ4	146433

IL-5 Ra	Q01344	3568
IL-10 Ra	Q13651	3587
Layilin	Q6UX15	143903
Leptin R	P48357	3953
Marapsin	Q9BQR3	83886
Mer	Q12866	10461
MMP-7	P09237	4316
P-Cadherin	P22223	1001
Prostasin	Q16651	5652
PSMA	Q04609	2346
SIGIRR	Q6IA17	59307
TGFb RIII	Q03167	7049
TF	P13726	2152
TWEAK	O43508	8742
ADAMTS13	Q76LX8	11093
Aggrecan	P16112	176
Angiotensinogen	P01019	183
B7-H1	Q9NZQ7	29126
BMPR-IA	P36894	657
BMPR-II	Q13873	659
Cadherin-11	P55287	1009
CD27	P32970	970
CD6	P30203	923
Ck beta 8-1	P55773	6368
CNTF	P26441	1270
DNAM-1	Q15762	10666
EMMPRIN	P35613	682
FLRG	O95633	10272
Follistatin-like 1	Q12841	11167

Fractalkine	P78423	6376
Galectin-1	P09382	3956
GITR L	Q9UNG2	8995
Granulysin	P22749	10578
IL-1 R3	Q9NPH3	3556
IL-15 R	Q13261	3601
IL-17E	Q9H293	64806
IL-32 alpha	P24001	9235
L1CAM-2	O00533	10752
LRIG3	Q6UXM1	121227
LRP-6	O75581	4040
MEPE	Q9NQ76	56955
Nectin-4	Q96NY8	81607
Periostin	Q15063	10631
Persephin	O60542	5623
Renin	P00797	5972
RGM-B	Q6NW40	285704
ROBO3	Q96MS0	64221
S100A8	P05109	6279
Siglec-7	Q9Y286	27036
Syndecan-3	O75056	9672
Thrombospondin-2	P35442	7058
Thrombospondin-5	P49747	1311
Tie-1	P35590	7075
ULBP-2	Q9BZM5	80328
ADAM8	P78325	101
ADAM12	O43184	8038
B7-H3	Q5ZPR3	80381
BMPR-IB	O00238	658

Cadherin-4	P55283	1002
Cadherin-13	P55290	1012
CD48	P09326	962
CD58	P19256	965
CD84	Q9UIB8	8832
CD99	P14209	4267
CD155	P15151	5817
CD229	Q9HBG7	4063
CEACAM-5	P06731	1048
CF XIV	P04070	5624
Cystatin A	P01040	1475
Cystatin B	P04080	1476
Cystatin E M	Q15828	1474
Desmoglein 2	Q14126	1829
DR3	Q93038	8718
ErbB4	Q15303	2066
ESAM	Q96AP7	90952
FGF-21	Q9NSA1	26291
Galectin-2	P05162	3957
Galectin-9	O00182	3965
ICOS	Q9Y6W8	29851
JAM-A	Q9Y624	50848
JAM-B	P57087	58494
Kallikrein 5	Q9Y337	25818
Midkine	P14174	4282
Pentraxin 3	P26022	5806
Pref-1	P80370	8788
Siglec-10	Q96LC7	89790
SLAM	Q13291	6504

SP-D	P35247	66421
Syndecan-4	P31431	6385
Testican 2	Q92563	9806
TIM-3	Q8TDQ0	84868
TLR4	O00206	7099
TRAIL	P50591	8743
ULBP-1	Q9BZM6	80329
