# Supplementary material for: Identification of Novel Plasma Biomarkers for Abdominal Aortic Aneurysm by Protein Array Analysis
Source: Biomolecules. 2022 Dec 12;12(12):1853. doi: 10.3390/biom12121853 (PMC9775419; doi:10.3390/biom12121853)
Supplement: Supplementary file 1 [file biomolecules-12-01853-s001.zip › Table S2.pdf]

Supplementary Table S2. Differentially expressed proteins between AAA patients and healthy controls with large area under the curve (AUC) values (AUC>0.85).

Gene Symbol	UniProt ID	AUC	95% confidence interval	P value	Sensitivity	Specificity
Clusterin	P10909	0.977	0.85-1.000	< 0.0001	100.00	90.00
MMP-7	P09237	0.955	0.816-0.997	< 0.0001	95.345	100.00
RGM-B	Q6NW40	0.918	0.766-0.985	< 0.0001	90.91	80.00
Epo R	P19235	0.9	0.742-0.977	< 0.0001	81.82	90.00
IL-1 RII	P27930	0.895	0.736-0.975	< 0.0001	77.27	100.00
Legumain	Q99538	0.882	0.719-0.968	< 0.0001	90.91	80.00
JAM-A	Q9Y624	0.864	0.696-0.959	< 0.0001	59.09	100.00
Cathepsin S	P25774	0.864	0.696-0.959	< 0.0001	72.73	100.00
ACE-2	Q9BYF1	0.859	0.691-0.956	< 0.0001	95.45	80.00
RANTES	P13501	0.859	0.691-0.956	< 0.0001	72.73	100.00
TPO	P40225	0.859	0.691-0.956	< 0.0001	81.82	80.00
NRG1-b1	Q02297	0.855	0.685-0.954	< 0.0001	86.36	70.00
OSM	P13725	0.85	0.680-0.951	< 0.0001	90.91	70.00

MMP-7: matrix metalloproteinase-7; RGM-B: RGM domain family member B; Epo R: erythropoietin receptor; IL-1 RII: interleukin-1 receptor type 2; JAMA: Junctional adhesion molecule A; ACE-2: angiotensin converting enzyme 2; RANTES: C-C motif chemokine 5; TPO: thrombopoietin; NRG1-b1: proneuregulin-1, and membrane-bound isoform; OSM: oncostatin M.
