# Supplementary material for: Identification of Novel Plasma Biomarkers for Abdominal Aortic Aneurysm by Protein Array Analysis
Source: Biomolecules. 2022 Dec 12;12(12):1853. doi: 10.3390/biom12121853 (PMC9775419; doi:10.3390/biom12121853)
Supplement: Supplementary file 1 [file biomolecules-12-01853-s001.zip › Table S3.pdf]

Supplementary Table S3. Differentially expressed proteins between AAM and AAN patients.

Gene Symbol	Uniport ID	Fold Change (AAM/AAN)	p-value
DAN	P41271	0.58	0.0009
FAS L	P48023	0.47	0.0015
Siglec-9	Q9Y336	0.59	0.0008
ANG-2	O15123	1.67	0.0028
ULBP-2	Q9BZM5	2.00	0.0024
CD84	Q9UIB8	1.99	0.0007
PIGF	P49763	2.29	0.0020
Dtk	Q06418	1.53	0.0001
E-Cadherin	P12830	0.49	0.0098
CD40	P25942	0.55	0.0121
FGF-21	Q9NSA1	1.98	0.0060
GCP-2	P80162	1.63	0.0034
IL-13 R1	P78552	0.55	0.0074
Prolactin	P01236	0.38	0.0382
DLL1	O00548	2.08	0.0059
CD23	P06734	1.68	0.0137
Cadherin-13	P55290	1.60	0.0135
Syndecan-4	P31431	1.86	0.0065
EGF	P01133	2.19	0.0059
FABP2	P12104	1.69	0.0216
PECAM-1	P16284	1.61	0.0295
VEGF-C	P49767	0.64	0.0141
EpCAM	P16422	0.47	0.0122
ICOS	Q9Y6W8	2.38	0.0423
IL-13 R2	Q14627	0.45	0.0257
b-NGF	P01138	1.97	0.0367

CEACAM-5	P06731	2.31	0.0295
ErbB4	Q15303	1.55	0.0375
TRAIL R4	Q9UBN6	0.63	0.0446
TIM-1	Q96D42	1.87	0.0411
VEGF-D	O43915	1.53	0.0458
